# Research on the Rule of Explosion Shock Wave Propagation in Multi-Stage Cavity Energy-Absorbing Structures

**DOI:** 10.3390/ma16134608

**Published:** 2023-06-26

**Authors:** Shihu Chen, Wei Liu, Chaomin Mu

**Affiliations:** 1 Pan Er Mine of Huaihe Energy Group, Huainan 232088, China; 2 Institute of Engineering Safety and Disaster Prevention, Hohai University, Nanjing 210098, China; 3 State Key Laboratory of Mining Response and Disaster Prevention and Control in Deep Coal Mines, Anhui University of Science and Technology, Huainan 232001, China

**Keywords:** explosion shock wave, explosive flame, cavity structure, propagation law

## Abstract

The propagation laws of explosion shock waves and flames in various chambers were explored through a self-built large-scale gas explosion experimental system. The propagation process of shock waves inside the cavity was explored through numerical simulation using Ansys Fluent, and an extended study was conducted on the wave attenuation effect of multiple cavities connected in a series. The findings show that the cavity’s length and diameter influenced the weakening impact of shock waves and explosive flames. By creating a reverse shock wave through complicated superposition, the cavity’s shock wave weakening mechanism worked. By suppressing detonation creation inside the cavity, the explosive flame was weakened by the cavity’s design. The multi-stage cavity exhibited sound-weakening effects on both shock waves and explosive flames, and an expression was established for the relationship between the suppression rate of shock force and the number of cavities. Diffusion cavities 35, 55, 58, and 85 successfully suppressed explosive flames. The multi-stage cavity efficiently reduced the explosion shock wave. The flame suppression rate of the 58-35 diffusion cavity explosion was 93.38%, whereas it was 97.31% for the 58-35-55 cavity explosion. In engineering practice, employing the 58-58 cavity is advised due to the construction area, construction cost, and wave attenuation impact.

## 1. Introduction

Gas explosions pose a danger to the production safety of coal mines [1,2]. Gas explosions produce shock waves and flames that travel into underground tunnels, severely damaging the people and equipment below. Shock waves from a coal mine gas explosion can kill people and damage tunnels and equipment. At the same moment, a big volume of coal dust is lifted and explodes, resulting in more destructive force. In addition, a large amount of harmful gases generated after the explosion can cause poisoning and death of personnel. The force of gas explosions can be somewhat diminished using explosion suppression devices. As a result, passive explosion suppression technology—which utilizes explosion-suppressing water bags and rock powder sheds—is widely employed in coal mines. An explosion suppression zone is created within a specific distance of the roadway when a gas explosion occurs because the shock wave destroys the water bag and rock powder shed. After being used once, the water bag and rock powder shed lose effectiveness and are powerless to stop repeated explosions. Therefore, addressing the issue of multiple explosion suppression and attaining consistent and dependable suppression during numerous explosions is practically significant for the underground safety of coal mines.

There are now two types of studies on coal mine gas explosion suppression technology. The first kind of technology is passive explosion suppression. Technology for actively suppressing explosions is the second category. Passive explosion suppression technology is primarily accomplished by dispersing explosive suppression powder or absorbing materials to reduce the intensity of explosion flames and shock waves. Through experiments, Liu et al. [3] investigated the effects of rock powder, water, and ABC dry powder on suppressing explosions. The findings revealed that while water and rock powder effectively suppressed shock waves and the Hull explosion flame of coal powder secondary explosion, ABC dry powder had the most effective overall suppressive effect. In order to undertake explosive suppression tests, Wang et al. [4] created Mg (OH)_2_/NH_4_PO_4_ composite dry powder (CDP) with various mass ratios. The findings demonstrated that CDP substantially impacted lowering the KG, Tmax, and Vmax of methane explosion. On a small-scale experimental platform, Luo et al. [5] confirmed the inhibitory capacity of BC powder on hydrogen/methane premixed gas explosions. The findings revealed that the ideal inhibitory concentration for BC was 200 g/m^3^. Experimental research on the NaHCO_3_ water mist’s ability to prevent methane explosions was carried out by Wei et al. [6]. The suppression mechanism of NaHCO_3_ water mist was examined using a kinetic model. The outcomes demonstrated that the explosive’s composition, equivalent, and concentration impacted the suppressing effect. Fan et al. [7] analyzed the effect of NaHCO_3_ powder on premixed flames through experiments. The results showed that when the particle size was large, thermodynamics controlled the inhibition mechanism, mainly by physical effects. When the particle size was small, the inhibition mechanism was controlled by kinetics, and chemical reactions dominated. Using a synchronous thermal analyzer, Zhao et al.’s research [8] on the inhibitory effect of ABC dry powder on methane/coal powder explosion revealed that ABC powder raised the initial temperature of the coal powder thermal decomposition and significantly decreased the thermal decomposition rate, heat release, and maximum heat flow rate. Using fluid mechanics and thermal analysis theory, Song et al. [9] conducted a numerical simulation study on the impact of rock powder on gas explosion suppression. The results revealed that when the amount of rock powder was less than 12 kg/m^3^, the flame in the pipeline could not be put out, and when Ningdu was 36 kg/m^3^, the overpressure decreased by 40%, and the flame peak speed decreased by 50%. In a wholly enclosed visual container, Jiang et al. [10] investigated the inhibitory impact of ultrafine water mist on methane explosions with methane concentrations of 6%, 11%, and 13%. Adding water mist lowered the maximum explosive overpressure, pressure increase rate, and flame propagation speed, according to the results. Through numerical modeling, Cao et al. [11] investigated the inhibitory mechanism of ultrafine water mist on methane explosion. The findings indicated that heat exchange occurred mainly in the reaction zone and that ultrafine water mist successfully suppressed methane explosion. Pei et al. [12] used a self-built water mist suppression device to perform several experimental tests on the suppression of methane explosions by water mist containing sodium chloride additions. The findings demonstrated that sodium chloride-containing water mist effectively inhibited methane explosions, primarily because the sodium chloride increased the synergistic impact of physics and chemistry. According to the properties of foam ceramics, Zhang et al. [13] investigated the coupling mechanism of foam ceramics to gas explosion flame and shock wave. The findings demonstrated that the primary variables influencing energy absorption and dissipation were the distinctive features of the porous structure. Through experimentation, Shao et al. [14] investigated the suppression impact of metal foam on gas explosions. The findings demonstrated that foam copper suppressed explosions more effectively at 6, 7, and 8 KPa starting pressures when placed near the ignition end. The results of a systematic study by Zhou et al. [15] on the effects of mesh aluminum alloy (MAA) and aluminum velvet (AV) on the explosion reaction of combustible gases revealed that these materials had a dual effect of promoting and suppressing explosions. The results also revealed that the primary function of explosion suppressants was determined by the nature of the combustible gas rather than the shape of the explosion suppressor material. Through experimental and computer simulations, Cheng et al.’s [16] study of the inhibitory impact of metal wire mesh on gas explosions revealed that metal wire mesh may efficiently reduce the flame’s temperature when premixed flames spread via a pipeline. The attenuation rate increased to 79% with the addition of three layers of 60 mesh metal wire mesh. Using a custom-made experimental apparatus, Sun et al. [17] investigated the effectiveness of porous materials in suppressing explosions. The findings demonstrated that foam ceramic and metal mesh materials had particular pressure-reducing and flame-retardant qualities. Metal mesh has excellent resistance to impact damage. However, it has a weak flame-retardant effect. Foam ceramic has a weak resistance to impact damage but an excellent flame retardant effect. In conclusion, passive explosive suppression technology may reduce the impact of gas explosions and explosion flames to a certain amount, but it often fails after one action and cannot withstand repeated explosions.

In order to regulate the explosion suppression device and spray explosion suppressants, active explosion suppression technology primarily employs high-precision sensors to record explosion information. Using autonomous spraying experimental equipment, Jiang et al. [18] looked into the inhibitory effects of nitrogen and ABC powder on methane explosion. The outcomes demonstrated that nitrogen and ABC powder spraying significantly reduced the overpressure of explosions and the flame propagation speed. The findings of an experiment by Chen et al. [19] using SiO_2_ powder to suppress a methane explosion indicated that SiO_2_ had a robust inhibitory impact on gas explosion flames, lowering the peak pressure and flame velocity by more than 40%. Yang et al.’s [20] experiment looked at how methane-oxidizing bacteria in ultrafine water mist affected methane explosions. According to the findings, fine water mist spraying effectively suppressed explosions, and methane-oxidizing bacteria had a part to play in the methane explanation, which enhanced the fine water mist’s suppression of the explosions’ impact. The findings of a 20 L spherical experimental setup utilized by Luo et al. [21] to investigate the inhibitory impact of CO_2_ and ABC powder on mine gas explosions revealed a synergistic effect between CO_2_ and ABC powder. The findings of a study by Zhao et al. [22] on the use of N_2_/APP to suppress fires and explosions caused by methane and coal dust in vertical pipes revealed that the system could successfully stop the spread of explosive flames caused by methane and coal dust. Li et al. [23] obtained the characteristics of explosion flames and explosion pressure through experiments by changing the equivalence ratio and water mist density. They analyzed the physical and chemical mechanisms of water mist-suppressed explosions. The results indicated that as the concentration of water mist increased, the average flame velocity, explosion peak overpressure, peak pressure rise rate, and positive pressure impulse all monotonically decreased. Jiang et al. [24] showed that fine water mist above 800 g/m^3^ could successfully inhibit detonation and ultimately led to flame extinction by using a sensor method to detect the spectrum signal of the explosion radiation to suppress methane explosions. To lessen the harm caused by gas explosions, Lu et al. [25] investigated using nitrogen gas to stop the spread of explosions in horizontal pipes. The findings demonstrated that nitrogen gas spraying might stop explosions from spreading throughout the pipeline when the nitrogen pressure rose beyond 0.3 MPa. An active gas explosion flame detection system was created by Lu et al. [26] and was used to automatically identify flames and spray extinguishing chemicals following a gas explosion. The findings showed that using ABC dry powder successfully put out explosion flames and that raising nitrogen pressure lowered the concentration of flammable gases in the pipeline. Using tests, Wang et al. [27] investigated the inhibitory impact of water mist containing KCL and N_2_ on methane explosions. The results revealed that CO_2_ inhibited the flame temperature better than water mist, while the water mist containing KCL and KCL inhibited shock wave overpressure and flame velocity more significantly. Active explosion suppression technology uses sensors to collect explosion information and a control system to direct the injection system to generate an explosive suppression region. It has the power to suppress many explosions, but it demands high system stability and a high cost, making it difficult to popularize. As a result, robust, dependable, and comprehensive explosion suppression technology is critical for coal mine safety.

There are several varied cross-sections in coal mine underground tunnels and mining processes. Therefore, considering the absolute engineering quantity and efficiency, the best length is 500 mm. The cavity’s length and diameter significantly influence the explosion shock wave and flame, even leading to the explosion being enhanced. As a result, this research can help direct coal mining. 

Using self-built large-scale explosion experimental equipment, the authors of this paper conducted explosive suppression tests on straight pipes and cavities 58, 55-35, 58-35, and 85-35. Ansys Fluent was used to investigate the shock wave propagation patterns in cavities 58-58 and 58-58-58, 58-58-58-58, and 58-58-58-58-58. The wave suppression effects of various types of cavities and the propagation laws and processes of shock waves in various cavities were computed. The best form of the cavity with the best explosion suppression effect was summarized, as was the link between the shock wave suppression rate and the number of cavities. This paper provides a reference for the future building of underground tunnel explosion suppression systems in coal mines.

## 2. Experimental Design

### 2.1. Gas Explosion Experimental System

**Figure 1:**
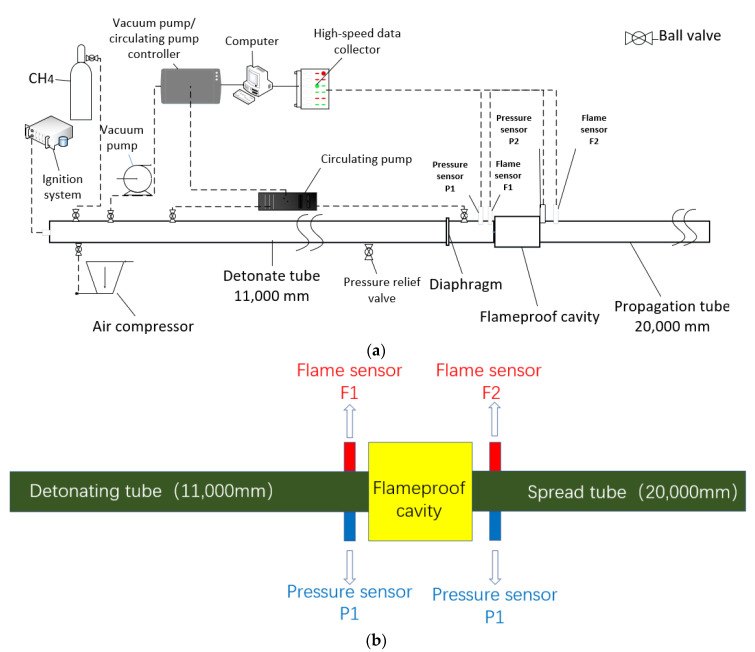
Gas explosion experimental system schematic diagram, (**a**) the system’s general schematic diagram, (**b**) the schematic diagram of the positions of pipelines, cavities, and sensors.

**Figure 2:**
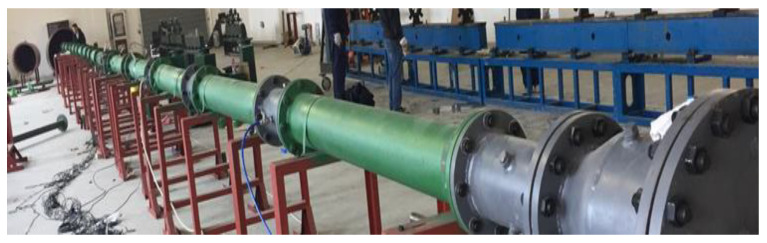
Field schematic diagram of the experimental setup for gas explosions.

We designed and built a large-scale gas explosion experimental apparatus with a diameter of 200 mm and a length of 36,000 mm. The experimental system comprised a gas distribution subsystem, a pipeline subsystem, an igniting subsystem, and a data-collecting subsystem group. Figure 1 depicts the schematic diagram. Figure 2 depicts pictures taken on-site. Figure 1a depicts the system’s general schematic diagram. Figure 1b shows the schematic diagram of the positions of pipelines, cavities, and sensors. Steel pipes with a wall thickness of 100 mm and an inner diameter of 200 mm were used in the pipeline subsystem, separated into detonating, connecting, and propagation pipes. The detonating tube was 11 m long, the connecting tube was 5 m long, and the propagation tube was 20 m long. The gas distribution subsystem comprised an air compressor, vacuum pump, gas cylinder, circulating pump, and electronic pressure gauge. The ignition subsystem comprised an ignition controller, power supply, electric fuse, and electrodes. The data collection system consisted primarily of pressure sensors, flame sensors, high-speed data-collecting instruments, and data-processing software. The ignition energy was 10 J, and the pressure sensor’s range was 0–3 MPa. The flame sensor had a maximum sampling rate of 20 MSPS and an accuracy of 0.1% FS. The strength of the gas explosion was affected by different ignition energies. According to Emmanuel et al. [28], an ignition energy of 10 J is preferable for gas explosion studies. Furthermore, Niu et al. [29] used a 10 J ignition energy in tests with a 9.5% gas concentration. Therefore, 10 J was used as the ignition energy in this research.

The following is the specific experimental procedure:

(1) Multiple systems were connected, and the instrument was debugged to restore it to regular operation;
(2) The diaphragm was inserted between the detonating tube and the connecting tube and seal with a sealing ring;
(3) A gas distribution subsystem was used to arrange the gas, with a concentration of 9.5% in the detonating tube;
(4) For 20 min, the circulating pump was used to circulate the gas in the pipeline;
(5) The collection subsystem was debugged to the collection state;
(6) For ignition, the ignition subsystem was used;
(7) To preserve the acquired data, a data-processing system was used.

### 2.2. Plan for Single-Stage Cavity Experiment

Explosion shock waves and explosion flames are the primary causes of gas explosion damage. As a result, understanding the development law of explosion shock waves and flames has practical implications for preventing and managing gas explosion disasters. Previously, our study group completed systematic research on the suppression laws of explosive shock waves in eight different types of cavities [30]. However, there is a dearth of systematic research on explosive flames. As a result, this study analyzed the rules of explosive flame development in eight different types of cavities. The authors of this article created eight cavity models of varying sizes. Table 1 shows the particular dimensions of the cavities.

The cavity names are shortened for clarity, as indicated in Table 2.

### 2.3. Plan for Multi-Stage Cavity Experiment

**Figure 3:**
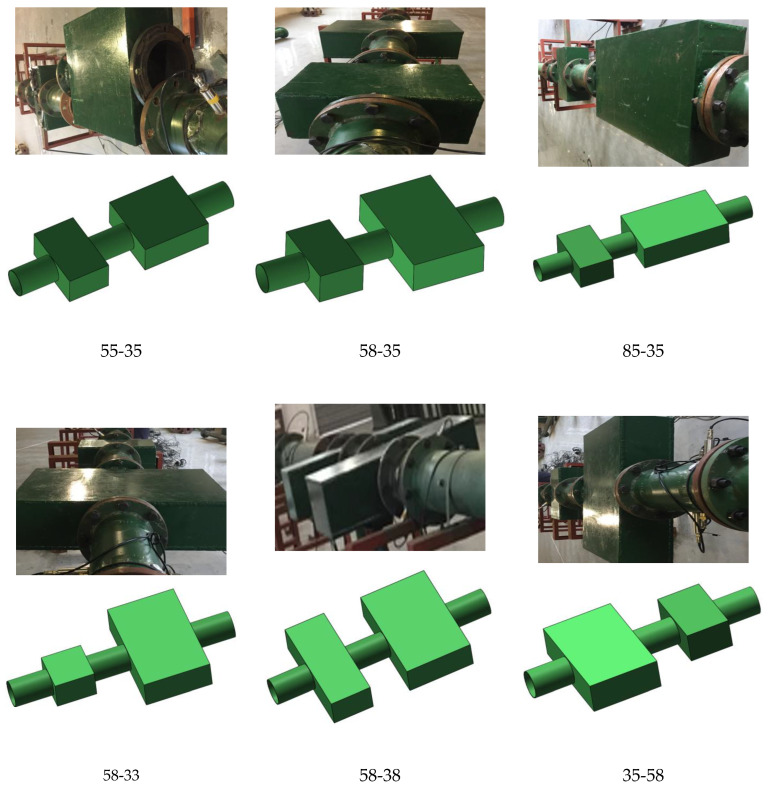

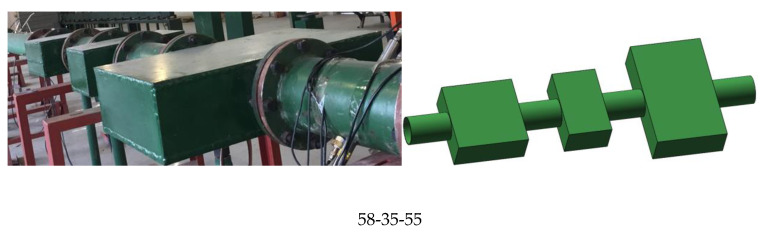
Multi-stage combined cavities.

Experiments on two-stage and three-stage combination cavities were carried out to explore the suppression impact of multi-stage combination cavities on gas explosion shock waves and flames. Several experiments were carried out by combining cavities of various sizes. Using the secondary combination cavities 55-35, 58-35, 85-35, 58-33, 58-38, and 35-58, combination tests were performed. The three-stage combined cavity combination was 58-35-55. Figure 3 is a snapshot of the experimental room.

## 3. Experimental Results and Analysis

The shock wave’s peak overpressure indicates the most significant breaking pressure and defines the suppression rate of the shock wave $\alpha =\frac{P_{1}-P_{2}}{P_{1}}$. The flame intensity is defined by the continuous light intensity gathered by the flame sensor, which is numerically equivalent to the region contained by the relationship curve between continuous light intensity and time, and determines the explosion flame suppression rate $\beta =\frac{F_{1}-F_{2}}{F_{1}}$.

### 3.1. Explosion Shock Wave and Flame Evolution Rule in Straight Pipes

**Figure 4:**
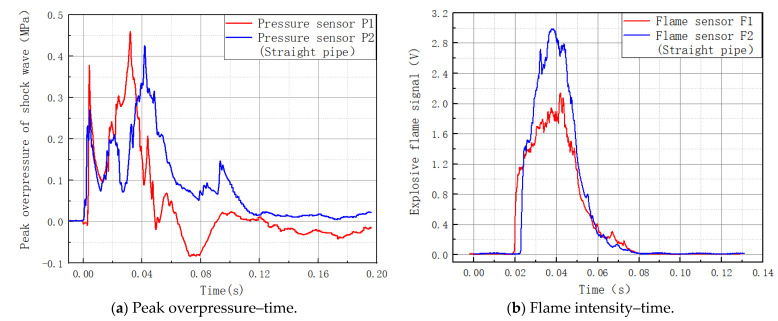
Straight pipe explosion shock waves and flames.

The straight tube experiment was used as a control experiment for the cavity experiment, with the pressure sensor and flame sensor positions remaining unchanged and the cavity replaced with a straight tube. Figure 4 depicts the connection between the shock wave overpressure and the time. Its maximal pressure suppression rate for shock waves was 8.11%. Figure 4 depicts the relationship curve between the flame front and the time. It had a flame suppression rating of −25.87%.

The gas–air combination was ignited at one end of the pipeline, and the explosion wave propagated from the ignition source to the unburned zone, which caused an increase in the flame and peak overpressure. At the same time, when a substantial quantity of energy was released, the surrounding gas expanded, and the temperature rose, causing the creation of precursor shock waves. The previous shock wave disturbed the unburned gas and caused it to flow through the pipeline; when it reached the F2 and P2 sensors, unburned gas remained. The unburned gas was instantly ignited, supplying energy for the explosion shock wave and increasing the flame and peak overpressure at the F2 and P2 sensor sites. 

### 3.2. The Flame Evolution Law of a Gas Explosion in a Single-Stage Cavity

**Figure 5:**
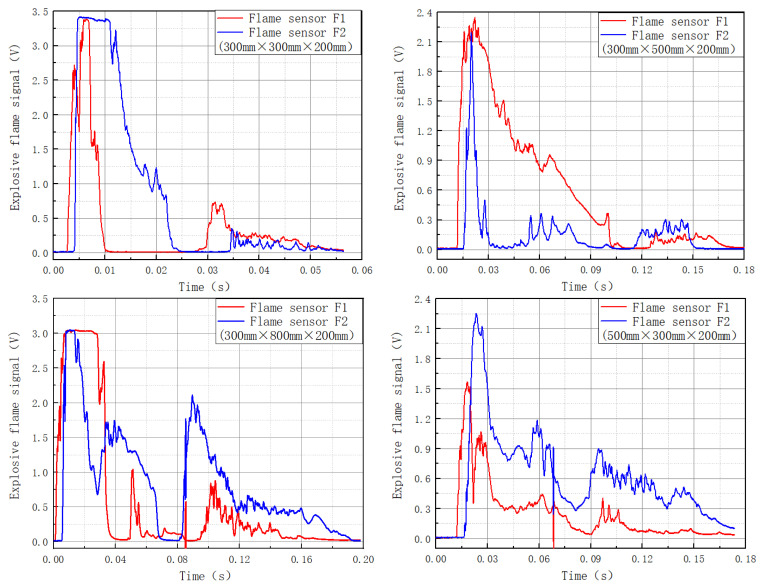

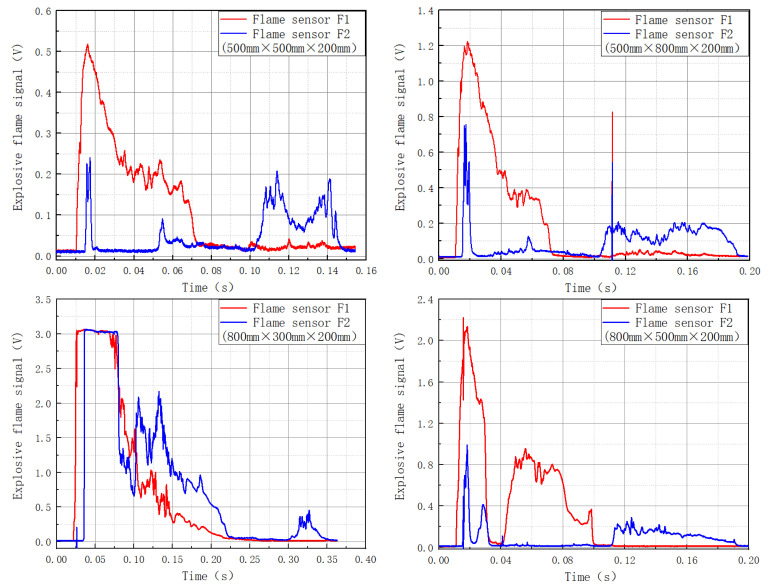
Explosion flame intensity of single-stage cavities of various sizes.

Figure 5 depicts the flame intensities of various-sized cavities. The suppression rate of an explosive flame by a single-stage cavity is shown in Table 3. Cavities 35, 55, 58, and 85 had a suppressive impact on explosive flames, whereas cavities 33, 38, 53, and 83 had an augmenting effect. Because the explosion shock wave traveled faster than the explosion flame, it entered the cavity first, subjecting the explosion flame to the combined effect of the cavity structure and the shock wave. 

The mechanism of the cavity and shock wave’s enhancement effect on the explosion flame was as follows: During the initial stage of ignition of the premixed gas and air, the mixture and gas began to burn, and the heat released by combustion caused the temperature of the mixed gas to rise rapidly and the volume to expand, resulting in the formation of a precursor shock wave. The front shock wave disturbed the unburned gas while propelling it ahead. After the shock wave reached the cavity, a complicated reflection superposition occurred, causing the shock wave’s velocity to drop. The explosion flame interacted with the shock wave inside the cavity, causing both the explosion flame and the shock wave to distort. When the shock wave collided with the explosion flame, the explosion flame became turbulent owing to instability, and combustion may have even changed into detonation.

The mechanism of the cavity and shock wave decreasing the explosion flame was as follows: Complicated emission superposition occurred when the explosion shock wave propagated to the cavity structure. The complex shock wave influenced the wavefront of the combustion wave and impacted the explosion’s continuation. Suppose the reflected waves formed in the cavity collide with the wave surface. In that case, the shock wave’s high flow velocity could dramatically reduce flame combustion speed and potentially cause the flame to die. This leads to the conclusion that the influence of the cavity on the explosive flame is dependent on the formation of the detonation event.

After the explosion flame passed through cavity 33, the primary explosion flame multiplied by 1.8, while the secondary flame front was weakened by 65.38%. Cavity 33 increased the flame front by 107.25% overall.

After passing through the cavity construction, the explosion flame simply decreased the primary flame energy surface and enhanced the secondary flame in cavity 35. Cavity 35 decreased the flame front by 6.7% overall.

After the explosion flame traveled through the cavity construction, the main flame front faded, the secondary flame front was strengthened, and the tertiary flame front was strengthened in cavity 38. Cavity 38 increased the flame front by 48.23% overall.

The total intensity of the explosion flame increased after going through the cavity construction in cavity 53. The flame showed local oscillation due to the non-uniformity of combustion.

The overall flame intensity in cavity 55 was reduced by 58.38% after the explosion flame traveled through the cavity construction. The flames were significantly suppressed by cavity 55.

The intensity of the explosion flame in cavity 58 dropped by 53.51% after traveling through the cavity construction. Cavity 58 effectively lowered the flame intensity by more than half.

The explosion flame oscillated dramatically after passing through the cavity structure in cavity 83, resulting in a secondary flame. Cavity 83 enhanced the explosive flame’s intensity by 149.19%.

**Figure 6:**
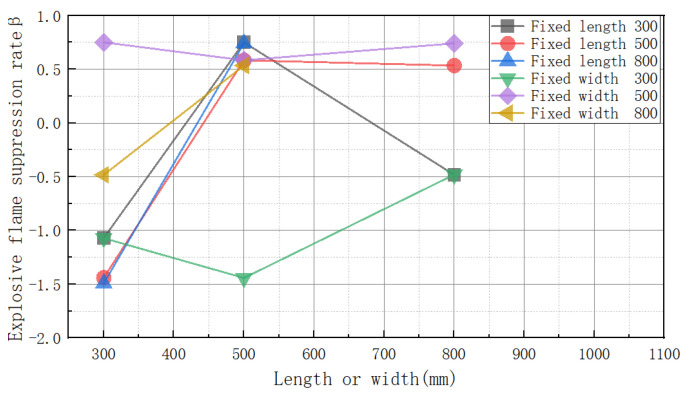
The effects of the length and width on the explosion flame suppression rate.

Figure 6 depicts the effect of cavity length and width on the suppression rate of explosive flames. When the length was set at 300 mm, the explosive flame suppression rates for 300 mm, 500 mm, and 800 mm widths were −107%, 75.12%, and −48.23%, respectively. It can be seen that the explosive flame suppression rate was optimum when the width was 500 mm. When the length was set at 500 mm, the explosive flame suppression rates for widths 300, 500, and 800 were −144%, 58.38%, and 53.51%, respectively. The effects of the cavities with widths of 500 mm and 800 mm were similar. When the length was 800 mm, the explosive flame suppression rates for 300 mm and 500 mm widths were −149.19% and 74.11%, respectively. The 500 mm visible width cavity offered the best explosion suppression effect. When the width was set to 300 mm, the explosive flame suppression rates for 300 mm, 500 mm, and 800 mm lengths were −107.25%, −144.31%, and −48%, respectively. The cavity had an amplification impact on the explosion flame when the length and width were minimal. The explosive flame suppression rates for 300 mm, 500 mm, and 800 mm lengths were 75.12%, 58.38%, and 74.11%, respectively, when the width was 500 mm. The explosion suppression effect was better when the width was 500. As a result, the capacity substantially impacted the suppression of explosive flames. When the width was fixed at 800 mm, the explosion flame suppression rates corresponding to 300 mm and 500 mm lengths were −48.23% and 58.51%, respectively. The volume of cavity 85 was the same as that of cavity 58, but the explosion suppression effect of cavity 58 was better than that of cavity 85. It can be seen that the explosion suppression effect was the result of the combined effect of the width and length.

Overall, the width of the cavity had a greater impact on the suppression rate of explosive flames than its length; for example, cavity 35 suppressed explosive flames, whereas cavity 53 enhanced them. Cavity 35 had an inhibiting influence on the explosive flames, but cavities 33 and 38 had an amplifying effect. It is clear that the broader the width, the stronger the impact of the explosion suppression. In conclusion, the influence of the explosive flames was determined by whether or not a detonation happened. Overall, the cavity width had a considerable effect on the pace of suppression of the explosive waves. 

### 3.3. Gas Explosion Shock Wave Evolution Rule in Multi-Stage Cavities

**Figure 7:**
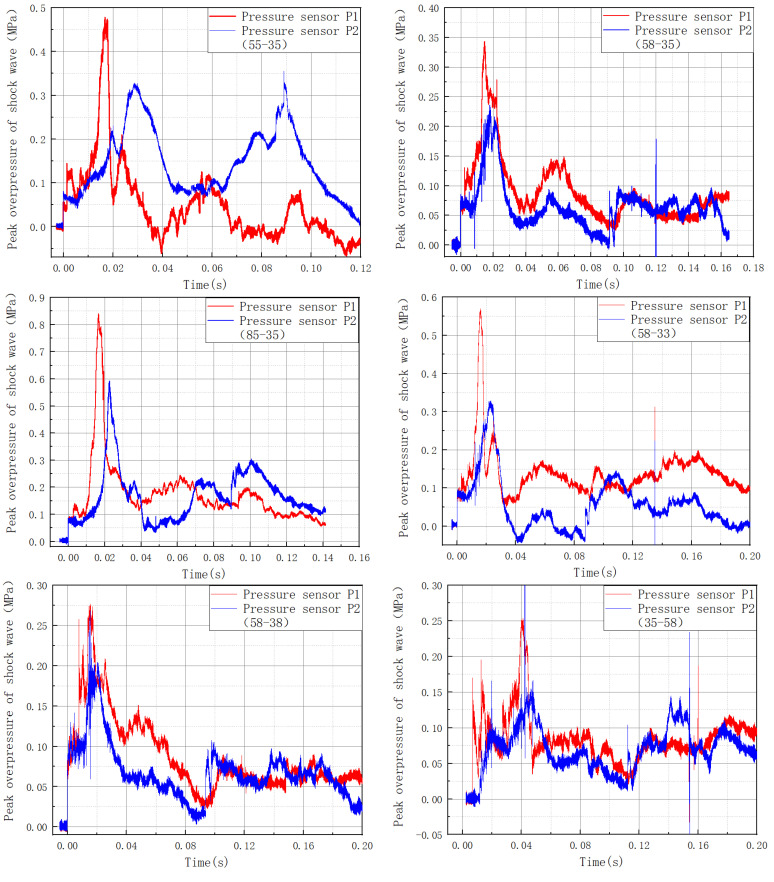

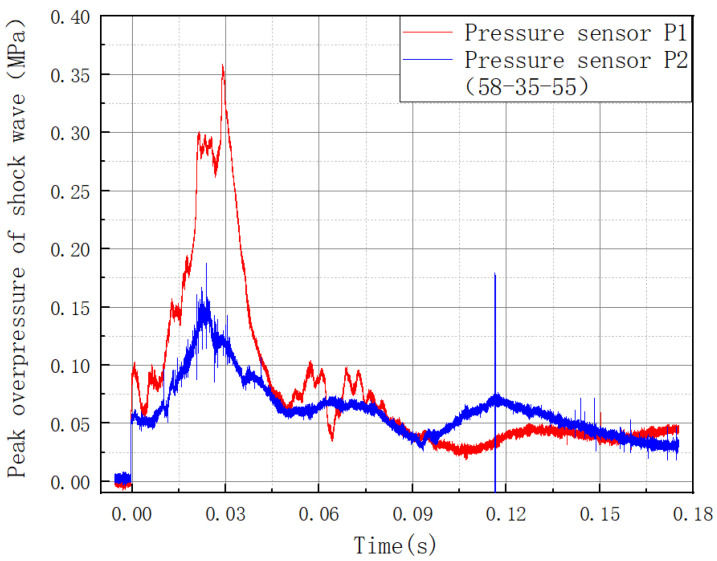
The multi-stage cavity shock wave’s peak overpressure.

The peak overpressure suppression rate of a single-stage cavity is displayed in Table 4 according to the results of the study group’s earlier work. Figure 7 depicts the shock wave’s peak overpressure data in a multi-stage combined cavity. The peak overpressure suppression rate of the multi-stage combined cavity shock wave is shown in Table 5. The combined cavities all dampened the explosion shock wave. The second cavities of the combination cavities 55-35, 58-35, and 85-35 were all 35. Because the single-stage cavity 35 enhanced the explosion shock wave, the wave attenuation impact of the combination cavity depended on the suppression effect of the single cavity on the shock wave. The inhibition rate of combination cavity 58-35 was 29.49%, and the inhibition rate of combination cavity 35-58 was 38.89%. Varying cavity installation sequences had varying suppression rates. As a result, the cavity with the highest suppression rate had a higher explosive suppression effect in the front. The peak overpressure suppression rate of the three-stage combination cavity was 54.74%, more significant than the rates of single cavities 58, 35, and 55. To summarize, the explosion suppression impact of the combination cavity was dependent on the effect of the single cavity.

### 3.4. The Law of Evolution of a Gas Explosion Flame in a Multi-Stage Cavity

**Figure 8:**
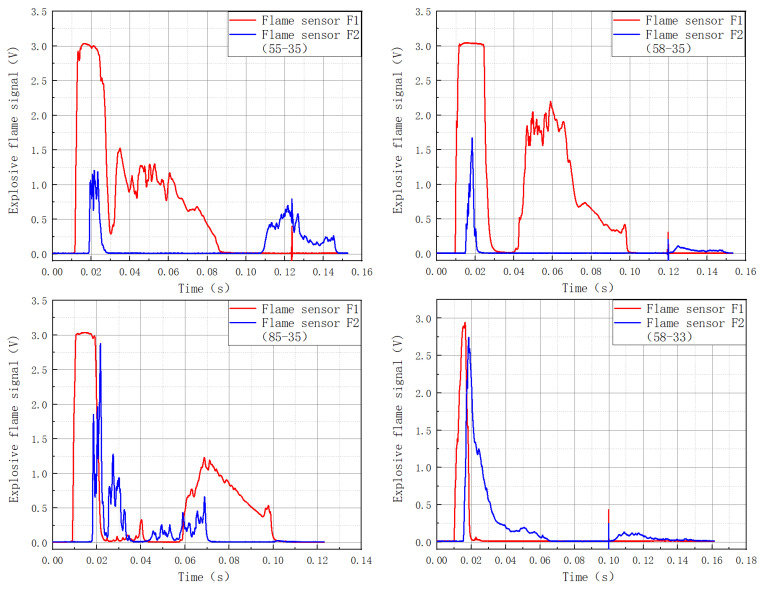

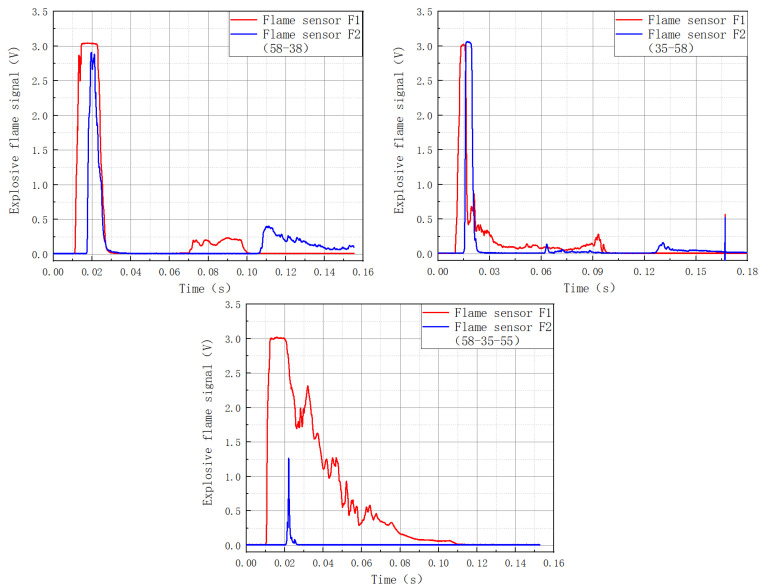
Intensity of flames in multi-stage combination cavities.

Figure 8 depicts the intensity of the flame in the multi-stage cavities. 

Table 6 depicts the flame suppression rate.

There was a significant secondary flame after the explosion flame passed through the 55-35 cavity. The natural flame attenuation of the F2 flame sensor was 87.1% when compared to the F1 flame sensor, while the secondary flame attenuation was 73.2%. The total intensity of the flame dropped by 79.78%.

The intensity of the explosion flame fell dramatically after passing through the 58-35 cavity, with a 94.7% attenuation. The secondary flame also fell dramatically, fading by 92.4%. 

The explosion flame attenuated after passing through the 85-35 cavity, although there was substantial oscillation, resulting in an overall flame attenuation of 71.80%.

After passing through cavity 58-33, the explosion flame intensified and a secondary flame formed, resulting in a 56.2% increase in flame intensity.

Following the passage of the explosion flame through the 58-38 cavity, the primary flame was attenuated by 57.5%, the secondary flame grew by 53.6%, and the overall attenuation was 42.73%.

Secondary and tertiary flames emerged after the explosion through the 35-58 cavity, and the total flame intensity was reduced by 34.63%.

The explosion flame was attenuated substantially and nearly vanished after passing through cavity 58-35-55, with an overall drop in the flame intensity by 97.3%.

The peak overpressure of shock waves was increased by diffusion cavities 3-3, 3-5, 3-8, 5-3, and 8-3. Cavities 5-5, 5-8, and 8-5 diffusion inhibited the shock wave’s peak overpressure. Because the cavity with a width of 300 mm and a length of 300 mm both had an enhancing impact on the shock wave’s peak overpressure, we shall address it here based on 500 mm. When the width was set at 500 mm, the suppression rate increased by 38.39% as the length expanded from 300 mm to 500 mm, and by 7.25% as the length extended from 500 mm to 800 mm. Therefore, considering the absolute engineering quantity and efficiency, the best length was 500 mm.

## 4. The Numerical Modeling of Shock Wave Propagation

### 4.1. Research Strategy for Numerical Simulation

It is advised to utilize cavity 58 in combinations since it had the best wave attenuation effect. Multiples of cavity 58 could not be created due to the experimental circumstances. As a result, numerical modeling was required to investigate the wave attenuation impact of numerous combinations of cavity 58. Numerical simulation investigations were conducted on the combinations of cavities 58, 58-58, 58-58-58, 58-58-58-58, and 58-58-58-58-58.

### 4.2. Geometric Modeling and Mesh Generation

The authors of this article created an 11 m detonation tube, a cavity, and a 20 m propagation tube for numerical simulation using Ansys Fluent numerical simulation software. A premixed gas of 9.5% methane and air was poured into the detonating tube. The set pressure monitoring points were 50 mm in front of and 50 mm behind the cavity. The ignition point was set at the end of the detonating tube, away from the cavity. The beginning circumstances were as follows: the temperature was 293 K, the premixed gas pressure was 1 e6Pa, and the speed was 0 m/s. The boundary requirement was no heat exchange between the pipeline and the wall surface inside the hollow.

**Figure 9:**
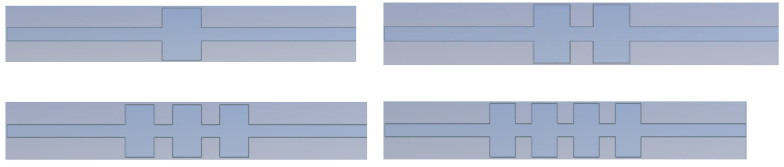

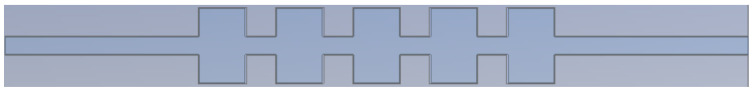
Flowchart for geometric modeling.

In order to take into account the calculation efficiency and the simulation accuracy, the explosive tube and the propagation tube are clearly divided in this paper, with a grid unit size of 0.05 m, while the cavity structure and connecting tube are clearly divided, with a grid unit size of 0.005 m. Figure 9 depicts the geometric modeling.

**Figure 10:**
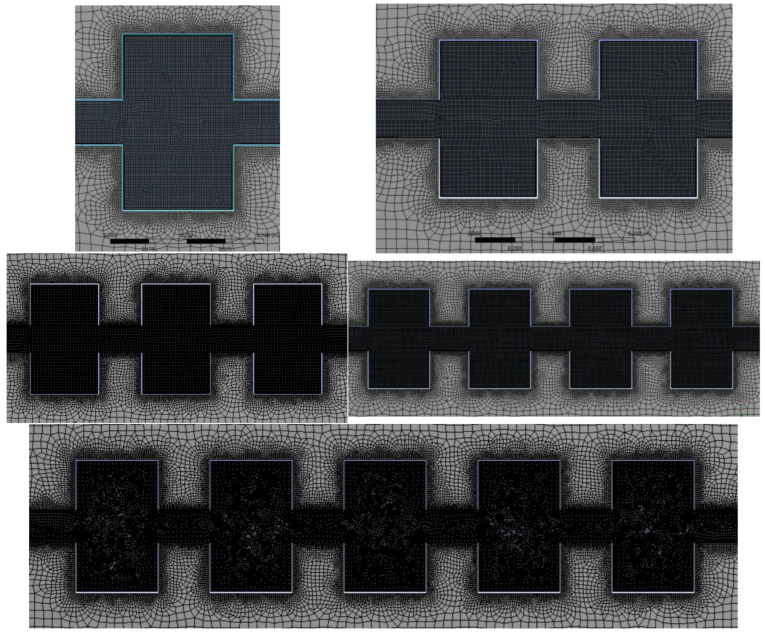
Schematic of grid division in geometric modeling.

Figure 10 depicts the grid division.

### 4.3. Mathematical Model

A mix of turbulence and chemical interactions caused the rapid and intricate processes of gas explosions and pipe combustion. The following presumptions were used when running the numerical simulations:

(1) It was assumed that the gas explosion process was a perfect mechanism for gas thermal expansion;
(2) It was assumed that the pipeline’s inner walls and the cavity’s interior surfaces were adiabatic, there was no heat exchange, the radiation heat release during the explosion shock wave propagation was disregarded, and the fluid–solid coupling effect between the inner wall and the shock wave was disregarded;
(3) It was assumed that in an explosion, the gas, and air were mixed equally, in line with Moore’s rule, and in a stationary condition prior to igniting;
(4) It was assumed that the shock wave created by the Mach rod was parallel to the inner wall and on a plane.

The authors of this paper utilized a two-step reaction model, which is a mature, high-precision, and high-reliability model with better accuracy, in order to increase the reliability and accuracy of the simulation results.

(1) $$\mathrm{Reaction}\;\mathrm{step}\;1:\;CH_{4}+O_{2}\to CO+H_{2}O+Q_{{}^{1}}$$

(2) $$\mathrm{Reaction}\;\mathrm{step}\;2:\;CO+O_{2}\to CO_{2}+Q_{2}$$

The gas dynamics model created in this article is based on compressible turbulent flow, encompassing the rules of mass conservation, momentum conservation, energy conservation, etc.

The chemical reaction rate of the reaction model’s induced reaction rate is:

(3) $$\omega_{\alpha }=-K_{1}\rho \exp(-E_{1}/RT)$$

Exothermic chemical reaction rate:

(4) $$\omega \beta =\left\{\begin{array}{l} -K2P^{2}\left\{\beta^{2}\exp(-E_{2}/RT)-{\left(1-\beta \right)}^{2}\exp(-\frac{E_{2}+q}{RT})\}\right.\\ 0,0<\alpha <1 \end{array}\right.,\alpha \leq 0$$

where *K*_1_ and *K*_2_ are the corresponding conventional coefficients of reaction rate, and α is the degree of induction progression and dimensionless quantity. β represents the rate of response progression. ρ is the density. The induced reaction’s activation energy is *E*_1_, and the exothermic chemical reaction’s activation energy is *E*_2_. *P* is the combined gas’s pressure, and P=ρRT. *R* stands for the gas constant, *T* for temperature, and *q* for the amount of heat released per mass of mixed gas.

Turbulence was chosen to be modeled by the RNG k−ε for a high Reynolds number. The equations for turbulent flow energy *K* and the turbulent flow energy dissipation rate ε must be solved to build Model k−ε. *K* is the formula for turbulent kinetic energy.

(5) $$\frac{\partial }{\partial t}\left(\rho k\right)+\frac{\partial }{\partial x_{i}}\left(\rho u_{i}k\right)=\frac{\partial }{\partial x_{i}}\left(\alpha_{k}\mu_{eff}\frac{\partial k}{\partial x_{i}}\right)+\tau_{ij}\cdot S_{ij}-\rho \varepsilon$$

The following is the equation for turbulent kinetic energy dissipation rate A:

(6) $$\frac{\partial }{\partial t}\left(\rho \varepsilon \right)+\frac{\partial }{\partial x_{i}}\left(\rho \varepsilon u_{i}\right)=\frac{\partial }{\partial x_{i}}\left(\alpha_{\varepsilon }\mu_{eff}\frac{\partial \varepsilon }{\partial x_{i}}\right)+C_{1\varepsilon }\frac{\varepsilon }{k}\tau_{ij}\cdot S_{ij}-C_{2\varepsilon }\frac{\rho \varepsilon^{2}}{k}$$

One way to represent the turbulent characteristic velocity u is as follows:

(7) $$u=k^{1/2}$$

One way to represent the turbulent characteristic length l is as follows:

(8) $$l=\frac{k^{3/2}}{\varepsilon }$$

The following is the expression of the connection between the mixed gas’s distinguishing characteristics and the turbulent combustion velocity *S* [31]:

(9) $$S_{t}=1.8u^{0.412}l^{0.196}S_{l}^{0.784}\nu^{-0.196}$$

(10) $$S_{ij}=(\tau_{ij}+\frac{2}{3}\rho k\delta_{ij})/(2\mu_{t})$$

(11) $$\mu_{eff}=\mu +\mu_{t}=\mu +\rho C_{\mu }\frac{k^{2}}{\varepsilon }$$

(12) $$\tau_{i}{}_{j}=\mu_{t}\left(\right.\frac{\partial u_{i}}{\partial x_{j}}+\frac{\partial u_{j}}{\partial x_{i}}\left.\right)-\frac{2}{3}\delta_{ij}\left(\right.\rho k+\mu_{t}\frac{\partial \mu_{j}}{\partial x_{j}}\left.\right)$$

where t is the time, i,j is the coordinate direction, $u_{i}$ is the gas explosion propagation velocity in the direction of the coordinate axis I, e is the particular internal energy, and $\mu_{t}$ is the turbulent viscosity coefficient. $\delta_{i}{}_{j}$ stands for the Kronecker operator, $C_{\mu }$ is considered to be 0.0845, $\alpha_{k},\alpha_{\varepsilon }$ is taken to be 1.39, $C_{1\varepsilon },C_{2\varepsilon }$ are taken to be 1.42 and 1.68, respectively, $S_{l}$ is the combustion velocity of laminar flames, and v is the kinematic viscosity.

### 4.4. Dependability Assessment of Numerical Simulation

**Figure 11:**
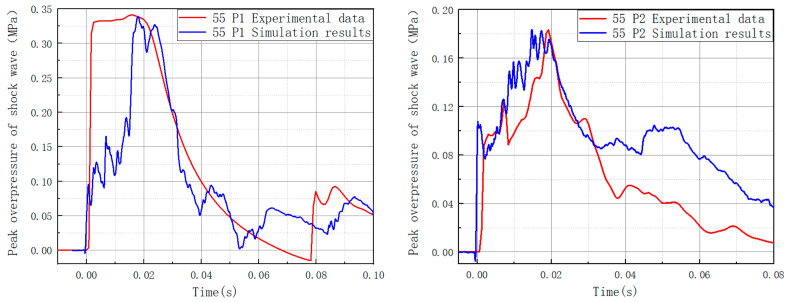
Comparison of the outcomes of numerical simulation and experiment.

The numerical simulation results were compared with the experimental data, as shown in Figure 11, to ensure that the numerical simulation was accurate. Because the peak value of the shock overpressure is an essential metric for describing the features of shock waves, we picked it as the comparison object. As can be observed, both the experimental and numerical simulation findings were close to the shock wave’s peak overpressure value, and both curves have a similar general tendency. The accuracy of the numerical simulation was established, and the following research on a numerical simulation expansion used this model and set of parameters. 

### 4.5. Numerical Simulation Results

#### 4.5.1. Law of Shock Wave Propagation in a Single-Stage Cavity

**Figure 12:**
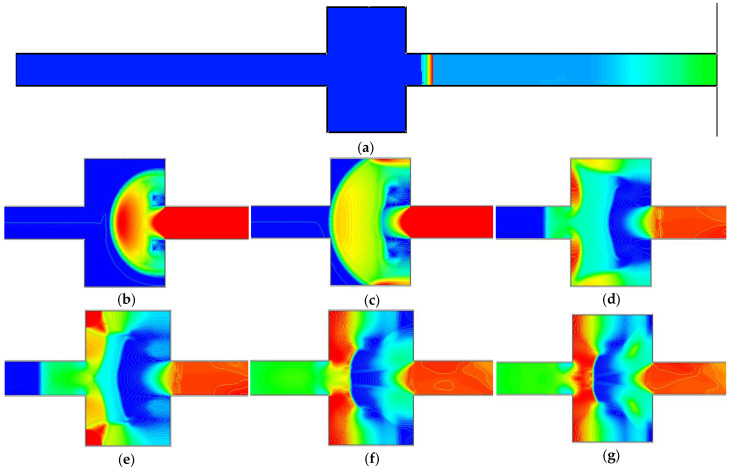

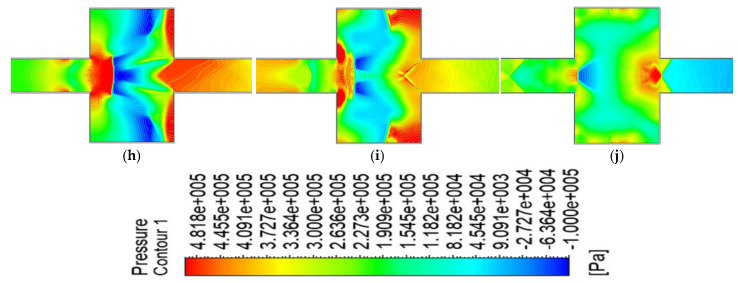
Comparison of experimental and numerical simulation results.

Figure 12 displays the simulation results for shock wave propagation during an explosion in cavity 58. The colors in Figure 12 reflect the pressure, and the pressure value may be established by comparing them to the color card.

The following are the steps in the dissemination process:

Before entering the cavity, as seen in Figure 12a, the shock wave traveled as a plane wave.

- As seen in Figure 12b, as the shock wave entered the diffusion cavity, the cross-section abruptly rose, and the plane wave transformed into a spherical wave;
- The high-pressure region of the shock wave shifted towards both sides of the cavity, as shown in Figure 12c, as the spherical wave met the obstruction of the inner walls on both sides and emitted superposition. Sparse waves were generated at the cavity’s center, and the shock waves were then reflected and stacked on the inner wall of the cavity, creating a Mach reflection, which created overpressure concentration areas on both sides of the cavity structure and caused the shock waves to propagate forward;
- As seen in Figure 12d, as the shock wave approached the cavity’s outlet, a section of it suddenly contracted and was blocked by the inner wall of the outlet, creating a reflected wave at the outlet, while the overpressure concentration area on both sides of the inner wall advanced;
- As seen in Figure 12e, when the right angle inner wall of the cavity blocked the overpressure concentration region on both sides of the shock wave inner wall, it underwent complex reflection superposition. It created a 45° overpressure reflection zone on the inner wall;
- The reflection overpressure concentration region shifted toward the cavity’s center, as seen in Figure 12f, as did the area on each side where it was concentrated;
- A reverse shock wave was created at the cavity outlet’s center, as shown in Figure 12g, and the reverse shock wave and forward shock wave canceled each other out to lessen the shock wave;
- This caused emission superposition and a high-pressure concentration area on both sides of the inner outlet wall, as shown in Figure 12h when the reverse shock wave hit the inner wall obstruction at the inlet;
- As seen in Figure 12i, overpressure concentration zones resurfaced on both sides of the inner wall, and the shock waves canceled each other out;
- As illustrated in Figure 12j, the shock wave experienced several reflections and overlays within the cavity, and the combined action resulted in the cavity having a good reduction impact on the explosion shock wave.

#### 4.5.2. Law of Shock Wave Propagation in a Multi-Stage Cavity

**Figure 13:**
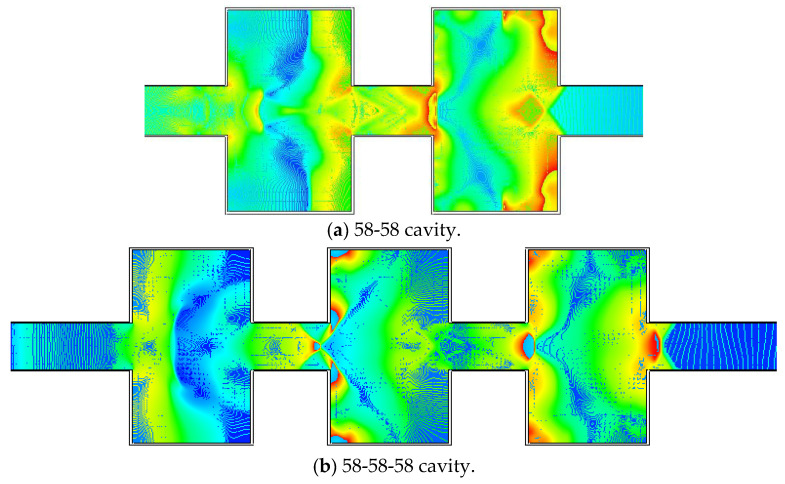

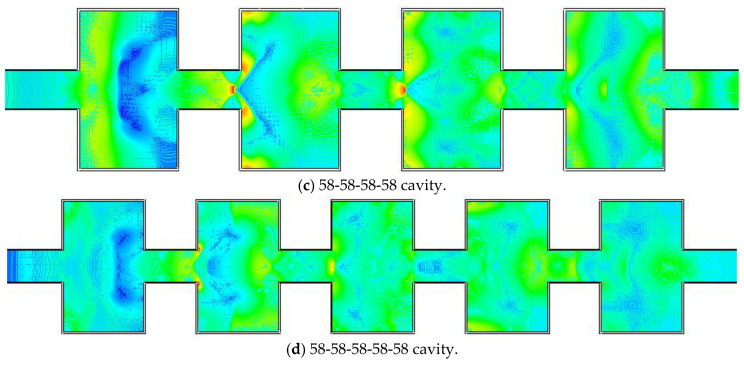
Comparison of experimental and numerical simulation findings.

The numerical simulation investigations of the 58-58 cavity, 58-58-58 cavity, 58-58-58-58 cavity, and 58-58-58-58-58 cavity were carried out. Figure 13 depicts the process of shock wave propagation. The shock wave propagation laws were comparable in each cavity. The propagation equation of shock waves in many cavities was more complicated, and the wave attenuation effect improved as the number of cavities grew.

The shock wave propagated in two cavities, as illustrated in Figure 13a, with the overpressure focused in the first. The leftover shock wave propagated in the second cavity after passing through the first cavity. The third cavity had fewer shock waves, as illustrated in Figure 13b, but the overall propagation pattern was comparable to that of a single cavity. The shock wave eventually decreased, as seen in Figure 13c, and there were fewer shock waves in the fourth cavity. As illustrated in Figure 13d, the shock wave progressively decreased, and the multi-stage cavity efficiently reduced the shock wave’s overpressure.

**Figure 14:**
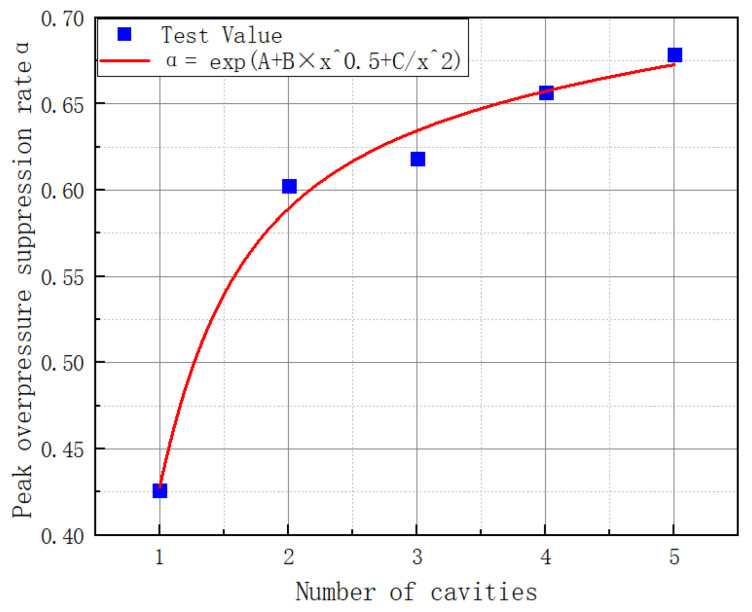
The relationship between the number of cavities and the shock wave suppression rate.

The numerical simulation results for the 58-58 cavity, 58-58-58 cavity, 58-58-58 cavity, and 58-58-58 cavity are shown in Figure 13. Figure 14 depicts the link between the number of cavities and the shock wave’s overpressure rate. Fitting the data through five points provides some credence [32,33,34]. Fitting yields the function equation between the number of cavities and the shock wave suppression rate:

(13) $$\alpha =e^{-0.52+0.06x^{0.5}-\frac{0.39}{x^{2}}}$$

In this formula, α is the shock wave suppression rate, and x is the number of cavities linked in a series.

R^2^ is a metric used to assess the quality of fitting. The closer the R^2^ is to one, the better the match. Figure 14 shows an R^2^ value of 0.98833. The root mean square deviation (RMSE) can mean the statistical dispersion of the data and is used to describe the correctness of the assessment value. The lower the value, the lower the statistical dispersion. In Figure 14, the RMSE is 0.03188.

The suppression rate of the shock wave overpressure in the 58-58 cavity rose by 14.98% when compared to the 58-58 cavity, by 1.6% when compared to the 58-58 cavity, by 3.83% when compared to the 58-58-58 cavity, and by 2.16% when compared to the 58-58-58-58 cavity. It can be shown that the shock wave suppression rate increased less for the 5-8 cavity, 58-58-58 cavity, 58-58-58 cavity, and 58-58-58 cavity. Given the available construction area and expense, it is recommended that the 58-58 cavity is built.

#### 4.5.3. Uncertainty Analysis

There may be significant uncertainties in the gas concentration, volume, detonation point, explosion distance, and explosion shock wave propagation process in actual explosion scenarios, resulting in discrepancies between actual explosions and experiments, empirical formulas, and numerical simulations.

(1) The uncertainty of the explosion parameters, including the TNT equivalent, gas concentration, explosion distance, ambient pressure, temperature, and site conditions were all considered;
(2) The unpredictability of the tunnel’s explosion shock wave flow field was considered;
(3) The collection device’s uncertainty was considered.

## 5. Conclusions

Using our own large-scale explosion experimental apparatus, we conducted experiments on straight tubes, and cavities 58, 55-35, 58-35, and 85-35. Ansys Fluent was used to investigate the shock wave propagation patterns in the 58-58 cavity, 58-58-58 cavity, 58-58-58 cavity, and 58-58-58 cavity. We evaluated the wave attenuation impact of several cavities, examined the shock wave propagation law and process in various cavities, and summarized the ideal cavity combination. The main conclusions are as follows:

(1) Cavities 35, 55, 58, and 85 had a suppressive impact on explosive flames, but cavities 33, 38, 53, and 83 had an augmenting effect. The interaction of shock waves and explosion flames led the explosion flame to become unstable, resulting in turbulence and even the occurrence of a combustion-to-detonation transition. When detonation occurred, the cavity had an amplifying impact on the explosive flame. The cavity had a suppressive impact on the explosive flames when the shock wave’s high flow velocity lowered the combustion speed of the flame and prevented detonation;
(2) The multi-stage cavity effectively lessened the explosion shock wave. The suppression rate of the 58-33 cavity with the best suppression of shock wave peak overpressure in the second cavity was 42.21%. With a suppression percentage of 54.74%, 58-35-55 had a good suppression impact on explosive shock waves. It was demonstrated through multi-cavity tests that linking numerous cavities in a series may substantially attenuate the explosion shock wave;
(3) Most combination cavities benefitted from weakening explosive flames. The 58-35 cavity had the best flame suppression effect on the two-stage cavity, with a 93.38% explosive flame suppression rate. The 58-35-55 three-stage cavity suppressed flames well, with a suppression rate of 97.31%. It can be seen that the 58-35-55 cavity had a good suppression effect on the explosion shock wave and explosion flame;
(4) According to the numerical modeling of the 5-8 single-stage cavity, the shock wave underwent complicated reflection and superposition, creating a reverse shock wave that effectively decreased the shock wave. The 58-58 cavity, 58-58-58 cavity, 58-58-58 cavity, and 58-58-58 cavity all reduced the explosion shock wave. The equation $\alpha =e^{-0.52+0.06x^{0.5}-\frac{0.39}{x^{2}}}$ represents the link between its inhibition rate and the number of cavities. The 58-58 cavity is suggested due to its construction space and expense.

## Figures and Tables

**Table 1:** Cavity parameters.

	Length (mm)	Width (mm)	Height (mm)	Experimental Photos	Schematic Model
1	300	300	200	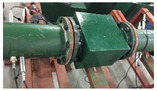	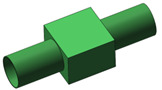
2	300	500	200	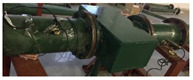	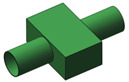
3	300	800	200	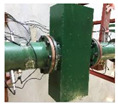	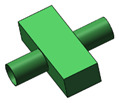
4	500	300	200	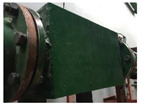	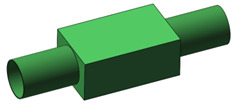
5	500	500	200	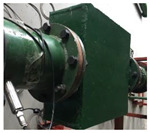	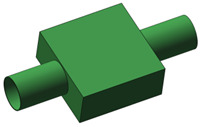
6	500	800	200	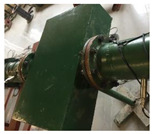	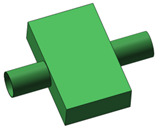
7	800	300	200	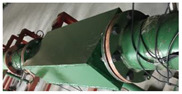	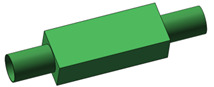
8	800	500	200	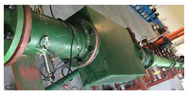	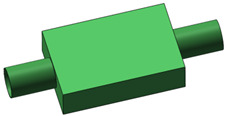

**Table 2:** Abbreviations of cavity names.

Cavity Size (Length × Width × Height)	Abbreviation
300 mm × 300 mm × 200 mm	33
300 mm × 500 mm × 200 mm	35
300 mm × 800 mm × 200 mm	38
500 mm × 500 mm × 200 mm	53
500 mm × 800 mm × 200 mm	58
800 mm × 300 mm × 200 mm	83
800 mm × 500 mm × 200 mm	85

**Table 3:** Rate of reduction of peak explosion flame pressure in a single-stage cavity.

Cavity Type	Area F1	Area F2	Rate of Explosion Flame Suppression
33	0.0207	0.0429	−107.25%
35	0.1009	0.0251	75.12%%
38	0.1121	0.1162	−48.23%
53	0.0422	0.1031	−144.31%
55	0.0173	0.0072	58.38%
58	0.0385	0.0179	53.51%
83	0.2547	0.2927	−149.19%
85	0.065	0.0167	74.11%

**Table 4:** Peak overpressure suppression rates of single-stage cavities.

	33	35	38	53	55	58	83	85
Rate of peak overpressure suppression	−19.55%	−16.91%	−14.69%	−26.91%	21.48%	45.27%	−32.60%	28.73%

**Table 5:** Peak overpressure suppression rates of multi-stage cavities.

	55-35	58-35	85-35	58-33	58-38	35-58	58-35-55
Pressure sensor P1(MPa)	0.4778	0.3432	0.8403	0.5689	0.2769	0.2535	0.3584
Pressure sensor P2(MPa)	0.3283	0.2356	0.5925	0.3288	0.2048	0.1549	0.1622
Rate of peak overpressure suppression	31.29%	31.35%	29.49%	42.21%	26.04%	38.89%	54.74%

**Table 6:** Rates of multi-stage combination cavities’ flame control.

	55-35	58-35	85-35	58-33	58-38	35-58	58-35-55
Flame sensor F1	0.0945	0.1088	0.0649	0.0188	0.0434	0.0283	0.0929
Flame sensor F1	0.0019	0.0072	0.0183	0.0293	0.0252	0.0185	0.0025
Flame suppression rate	79.78%	93.38%	71.80%	−55.85%	42.73%	34.63%	97.31%

## Data Availability

Not applicable.
